# Sidransky Syndrome—*GBA1*-Related Parkinson’s Disease and Its Targeted Therapies

**DOI:** 10.3390/ijms26073435

**Published:** 2025-04-06

**Authors:** Majdolen Istaiti, Gilad Yahalom, Mikhal Cohen, Volha Skrahina, Aliaksandr Skrahin, Jan Lukas, Arndt Rolfs, Ari Zimran

**Affiliations:** 1 Agyany Pharma Ltd., Jerusalem 9695614, Israel; joleen.istaiti@agyanypharma.com (M.I.); volha.skrahina@agyanypharma.com (V.S.); aliaksandr.skrahin@gmail.com (A.S.); arndt.rolfs@agyanypharma.com (A.R.); 2 Gaucher Unit, The Eisenberg R&D Authority, Shaare Zedek Medical Center, Jerusalem 9103102, Israel; 3 Department of Neurology and Movement Disorders Unit, Shaare Zedek Medical Center, Jerusalem 9103102, Israel; gyahalom@gmail.com (G.Y.); mikhalc@szmc.org.il (M.C.); 4 Rare Disease Consulting RCV GmbH, Leibnizstrasse 58, 10629 Berlin, Germany; 5 Translational Neurodegeneration Section Albrecht Kossel, Department of Neurology, University Medical Center Rostock, 18147 Rostock, Germany; jan.lukas@med.uni-rostock.de; 6 Center for Transdisciplinary Neurosciences Rostock (CTNR), University Medical Center Rostock, University of Rostock, 18147 Rostock, Germany

**Keywords:** Sidransky syndrome, *GBA1*-related Parkinson’s disease, pharmacological chaperones, Ambroxol, Gaucher disease, genetic testing

## Abstract

Sidransky syndrome represents a distinct variant of Parkinson’s disease (PD) that is linked to pathogenic variants in the glucocerebrosidase (*GBA1*) gene. This disorder exhibits an earlier onset, a more severe course, and a higher dementia prevalence compared to idiopathic PD. While the pathogenesis remains debated between loss-of-function and gain-of-function mechanisms, targeted therapies are emerging. Pharmacological chaperones (PCs), like high-dose Ambroxol, aim to mitigate enzyme misfolding—a primary driver of this disorder—rather than addressing metabolic deficiencies seen in Gaucher disease. Despite failed trials of substrate reduction therapies, current clinical trials with Ambroxol and other PCs highlight promising avenues for disease modification. This commentary advocates for increased awareness of Sidransky syndrome to advance diagnostic strategies, promote genetic testing, and refine targeted treatments, with the potential to transform care for *GBA1*-related PD and prodromal stages of the disease.

The first paper reporting a possible link between Gaucher disease (GD) and Parkinson’s disease (PD) had a hard time being published, as it was rejected six times before it was accepted to *QJM* in 1996 [1]. A key challenge at that time was the difficulty in fully understanding and integrating the concept of a real link—rather than a mere coincidence—between the rare genetic disorder and the second most common neurodegenerative disease, which contributed to its delayed acceptance. Later, with dedicated research and numerous contributions, most notably by Ellen Sidransky from the NIH, and particularly her meta-analysis from 2009 (which confirmed an odds ratio of 5.43 for each *GBA1* variant in PD patients compared to controls) [2], *GBA1*-related PD became an established entity with the following three unique features compared to idiopathic PD: an earlier age of onset, a more severe clinical course, and a higher likelihood of developing dementia [3]. Whilst *GBA1*-PD is now textbook knowledge, the underlying pathogenesis remains controversial, and the academic debate between haploinsufficiency (loss of function) and gain of function [4] has influenced the development of completely different treatment approaches. Interestingly, each of the schools was able to develop an animal model to justify the chosen therapeutic modality. Based on a mouse model that is homozygous for D409V, developed by Sardi and colleagues [5], Sanofi has made significant investments in using venglustat, a substrate reduction therapy (SRT) capable of crossing the blood–brain barrier (BBB) in phase I and phase II clinical trials for patients with *GBA1*-PD [6,7]. Other researchers supporting the gain-of-function explanation have used other animal models [3]—notably the fruit fly *Drosophila* [8,9]—to explore the use of pharmacological chaperones (PCs), mainly Ambroxol (an over-the-counter (OTC) cough medicine which has been shown to be a glucocerebrosidase-specific PC [10,11]), to treat both *GBA1*-PD and idiopathic PD (assuming that misfolding could also occur in the wild-type glucocerebrosidase enzyme) [10,11].

While the double-blind, placebo-controlled clinical trial of venglustat was being conducted as a multinational multicenter study and representatives of the company and/or some of their investigators were promoting its great potential at various medical conferences, we (AR, AZ and colleagues; [12]) published a viewpoint entitled “Substrate Reduction Therapy for *GBA1*-Associated Parkinsonism: Are We Betting on the Wrong Mouse?” The most important reason not to consider SRT for *GBA1*-PD is the basic fact that *GBA1* carriers do not accumulate glucosylceramide (the substrate) in the reticuloendothelial cells in the spleen, liver, and bone marrow, and certainly not in the dopaminergic neurons. If this were the underlying mechanism, then all carriers should have been patients, and we would have seen more patients with GD suffering from *GBA1*-PD than carriers. In reality, carriers of the non-N370S (also referred to as p.Asn409Ser) variants have a higher risk of developing PD than patients with GD, who have two mutant alleles and twice the level of the mutant enzyme [3,4]. Paradoxically, patients who had GD and PD were excluded from the clinical trial with venglustat. In February 2021, exactly one year after the publication of the article with the wrong mouse viewpoint, the clinical trial of venglustat *GBA1*-PD was stopped by Sanofi because venglustat actually ‘did not show any beneficial treatment effect compared to placebo’ [7]. Based on the information contained in this article, there appear to have been more adverse events, particularly severe treatment-emergent CNS-related complications, in the venglustat group than in the placebo cohort. This is not surprising, since *GBA1*-PD is not a manifestation or a reflection of the metabolic aspect of GD. It is not related to the glucocerebrosidase function, which hydrolyzes glucocerebroside (GC) in the lysosomes of macrophages, but it is related to the misfolding of the mutant enzyme in the endoplasmic reticulum (ER), leading to ER stress, to UPR (Unfolded Protein Response), a stress response mechanism aiming at reaching homeostasis. When this is not reached, it leads to various pathophysiological processes that promote inflammation, the aggregation of alpha-synuclein in dopaminergic neurons [8], and, ultimately, cell death (degeneration) and the development of *GBA1*-PD [13]. In addition to several studies demonstrating the UPR in animal models of GD and of *GBA1*-related PD, which are detailed in the previous references [3,4,8], the *GBA1* D409V knock-out mouse model shows markedly reduced GCase activity in the liver and brain, with glycosphingolipid accumulation, but not UPR. Moreover, unchanged dopaminergic neuron counts in the substantia nigra were detected, and no α-synuclein pathology or was observed in the nigrostriatal system [14]. Evaluations of *GBA1*-related PD post mortem brains have also revealed altered levels of UPR-associated proteins, BiP, CHOP, and HERP [15,16]. Accordingly, treatment should not be copied from the way we treat GD, but should aim to correct the misfolding and thereby stop any downstream consequences from using PCs. Indeed, the SRT approach has failed, and the attempt to deliver the human recombinant glucocerebrosidase enzyme replacement therapy (ERT) to the brain using an Adeno-associated virus (AAV)-based gene therapy (by direct injection to the cerebrospinal fluid (CFS) via the cisterna magnum) is unlikely to help either [17]. The addition of exogenous wildtype enzyme cannot correct the misfolding of the endogenous mutant glucocerebrosidase within the ER. These were not the only clinical trials based on the haploinsufficiency hypothesis—two others that have already been performed in human subjects (healthy volunteers or *GBA1* carriers with PD). These include the use of Magnetic Resonance Imaging (MRI)-guided low-intensity focal ultrasound (LIFU) to temporarily open the blood–brain barrier (BBB) while the *GBA1*-PD patients are receiving an intravenous infusion of imiglucerase (Cerezyme™; Boston, MA, USA) [18], and the second involves a new small molecule, an allosteric activator of the glucocerebrosidase that stimulates/increases the activity of either wild-type or mutant enzymes (LTI-291, Bial Pharmaceuticals, Portugal; [19,20]). As all the above-mentioned approaches, particularly those attempting to improve the delivery of the enzyme GCase into the brain tissue (and not merely to the exogenous CSF space), are at a very early stage of clinical development; time will tell whether our prediction is right or wrong.

The second approach assumes that gain of function is the reason for *GBA1*-PD, using PCs as a therapeutic intervention. Again, there are good animal models mentioned earlier, particularly *Drosophila*, and there are also on-going clinical trials using either high-dose Ambroxol, or several new drugs, in different stages of clinical development, including one formulation, that has recently completed phase 1 of the safety trial in Australia in healthy volunteers (https://gaintherapeutics.com/investors-media/news-events/ accessed on 26 March 2025).

Ambroxol is an expectorant that was developed by Boehringer-Ingelheim (Ingelheim, Germany) in the late 1960s/1970s and commercialized in 1978 [21]. Since then, it has been used as an OTC medication in many countries (except the USA). It was identified as a potential specific PC for GD by Maegawa and Mahuran back in 2009 [22] when they developed a thermal denaturation assay utilizing wild-type glucocerebrosidase to screen a library of 1040 commercially approved drugs. Originally, this search was for a PC to treat GD. After proving the concept in patients with type 1 GD [23], Narita and colleagues in Japan successfully applied this approach to treat patients with advanced neuronopathic GD (nGD) by conducting a well-designed investigator-initiated research (IIR) [24]. The actual demonstration of reversibility of several key manifestations of nGD, such as debilitating myoclonic epilepsy or inability to walk due to severe ataxia, has inspired us and others to use Ambroxol in patients with *GBA1*-PD, and even in non-*GBA1*-PD, hypothesizing that misfolding can occur to some degree also in individuals without mutations at the DNA level [10,11]. The advantages of repurposed drugs—whether identified by serendipity, such as acetylsalicylic acid, thalidomide or sildenafil [25], or more recently by data mining and computational drug discovery methodologies [26]—is twofold. The first advantage is the possibility to skip the stage of toxicology during the drug development, thereby shortening the time to market; the second—particularly for an OTC drug—is its availability, providing a high level of confidence regarding its safety, and allowing patients to receive it “off-label” with or without official monitoring. In the case of Ambroxol for nGD and PD, one cannot skip toxicology at all, because as a cough medicine it is typically given for a few days and in smaller doses (typically 150 mg/day), whereas for GD and PD it is a long-term administration (possibly lifetime) and the most widely used dose is several fold higher than the suggested OTC dose (most users and clinical trials are using 1200 mg/day or in children up to 25 mg/kg/day) [10,24]. The second advantage of access to a repurposed drug is indeed relevant to Ambroxol, and many patients with nGD, as well as those with *GBA1*-related PD or even idiopathic PD, have begun using it with satisfactory results without the need to wait for lengthy clinical trials [27]. Yet, many patients have not benefited from Ambroxol because their physicians did not recommend using unproven approach, or simply due to the fact that OTC medications are never reimbursed.

In an attempt to upgrade the level of evidence and thereby of international trust in Ambroxol, particularly for nGD and *GBA1*-PD, we have established an investigator-initiated drug registry which originally included 41 patients, receiving a daily dose between 75 and 1485 mg (median 435 mg, which is roughly three times the daily dose when used as a cough medicine) that is followed for a period of 1 to 76 months (median 19 months; [27]), and we are currently updating this report to include over 150 patients, with a longer follow-up and a higher mean dose of Ambroxol. Currently there are 4 on-going clinical trials with high-dose Ambroxol at different doses with different study designs (out of 14 active Phase 1–3 trials evaluating drug therapies for *GBA1*-PD registered on ClinicalTrials.gov, as of 31 January 2024), yet none of them is an FDA-guided trial which will open the door for the commercialization of Ambroxol for the two indications mentioned above [20].

While early data seem promising regarding the potential of Ambroxol to favorably impact the course of *GBA1*-PD [3,10,27], this drug—like any other pharmacological chaperon that may be developed for this indication—is unlikely to be equally effective for all different variants, as different mutations can lead to distinct folding defects or stability issues of the mutant misfolded proteins [28].

An earlier stage to the conduction of clinical trials is obviously the need to identify those individuals who are either at risk or have already developed *GBA1*-related PD. Here, we are facing a bigger challenge, which is the fact that there is very little awareness within the community of neurologists in general, and even among movement disorders specialists in particular of the need to perform a genetic testing, even to newly diagnosed patients with PD. In a comprehensive review of PD published recently in the *New England Journal of Medicine* (*NEJM*) [29], while there is a detailed discussion of genetic predisposition, Ambroxol is not mentioned even once, despite the four on-going clinical trials. At the most recent international congress of the Movement Disorders Society (MDS) in September 2024 in Philadelphia, the opening session included three lectures under the title of “Management of Early Parkinson’s Disease”—the two words “genetic testing” were not mentioned even once! This is all the more disappointing when we know that early therapy already at the prodromal stage, when available, may prevent or, at minimum, delay the onset of full-blown devastating PD [29]. Indeed, poor awareness is the number one characteristic of rare diseases. *GBA1*-PD falls within this category. While this is the most common genetic cause of PD, based on the latest information coming from the ROPAD study showing a 10.4% positive *GBA1* variant among 12,580 patients with PD [30], given the estimated number of patients in the USA suffering from PD being more than 1 million [31], this 10.4% falls within the definition of rare disease, which is less than 200,000 (https://www.fda.gov/patients/rare-diseases-fda accessed on 14 February 2025).

In order to increase awareness for this form of PD, which is clearly a distinct variant, with an early age of onset, existing in both carriers and patients with GD, from the prodromal phase (higher prevalence of autonomic manifestations) to a full-blown hypokinetic movement disorder, which is typically more severe with a shorter time of survival and a higher prevalence of cognitive impairment, and also Lewy body dementia, it seems appropriate to create a new eponym in medicine—Sidransky syndrome (Figure 1). The molecular diagnosis of mutation at the *GBA1* DNA level is a prerequisite for the diagnosis; hence, having the eponym would facilitate genetic testing at large for all newly diagnosed patients with PD, thereby serving the best interest of the PD population beyond *GBA1*. In parallel, Dr. Ellen Sidransky’s pioneering contributions in the field of association of Gaucher and Parkinson’s disease, spanning fundamental research to innovative therapies, have profoundly shaped our understanding of this entity, making it fitting that her name be associated with it. It is to be hoped that with greater awareness for Sidransky syndrome, we can further improve our scientific and medical understanding of the impact of glucocerebrosidase on Parkinson’s disease.

## Figures and Tables

**Figure 1:**
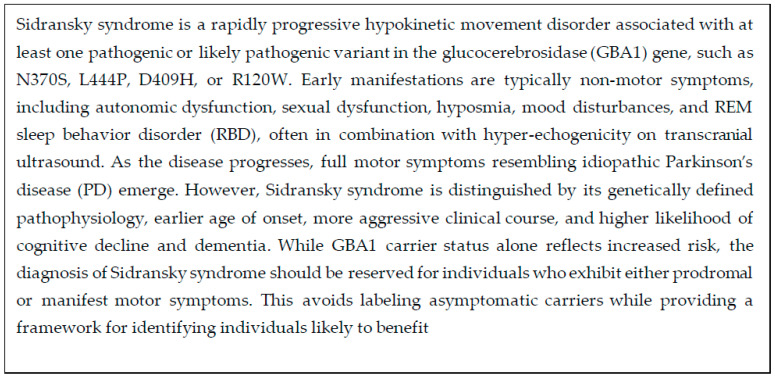
Sidransky syndrome.

## Data Availability

Not applicable.
